# Functional gene groups are concentrated within chromosomes, among chromosomes and in the nuclear space of the human genome

**DOI:** 10.1093/nar/gku667

**Published:** 2014-07-23

**Authors:** Annelyse Thévenin, Liat Ein-Dor, Michal Ozery-Flato, Ron Shamir

**Affiliations:** 1 Genome Informatics, Faculty of Technology and Institute for Bioinformatics, Center for Biotechnology (CeBiTec), Bielefeld University, Bielefeld 33615, Germany; 2 Blavatnik School of Computer Science, Tel Aviv University, Tel Aviv 69978, Israel; 3 IBM Research—Haifa, Mount Carmel, Haifa 3498825, Israel

## Abstract

Genomes undergo changes in organization as a result of gene duplications, chromosomal rearrangements and local mutations, among other mechanisms. In contrast to prokaryotes, in which genes of a common function are often organized in operons and reside contiguously along the genome, most eukaryotes show much weaker clustering of genes by function, except for few concrete functional groups. We set out to check systematically if there is a relation between gene function and gene organization in the human genome. We test this question for three types of functional groups: pairs of interacting proteins, complexes and pathways. We find a significant concentration of functional groups both in terms of their distance within the same chromosome and in terms of their dispersal over several chromosomes. Moreover, using Hi-C contact map of the tendency of chromosomal segments to appear close in the 3D space of the nucleus, we show that members of the same functional group that reside on distinct chromosomes tend to co-localize in space. The result holds for all three types of functional groups that we tested. Hence, the human genome shows substantial concentration of functional groups within chromosomes and across chromosomes in space.

## INTRODUCTION

Cellular processes involve multiple types of functional relations between genes, including protein–protein interactions, regulatory relations and co-expression. Substantial research has been carried out regarding the interplay between functionally related genes and their arrangement on the genome. The most dramatic evidence for non-random organization of co-functioning genes is found in prokaryotes, where genes, usually from the same functional family, are often arranged in operons (1,2). Genes in an operon reside consecutively along the genome and are governed by a common promoter. In contrast, most studied eukaryotes lack operons, with few exceptions, including nematodes (3) and drosophila, where operons tend to be dicistronic (4) (see (3) for a review).

Various computational studies utilized the availability of whole genome sequences to show that eukaryotic functionally related genes do tend to cluster. Hershberg *et* *al*. used network analysis methods to show that adjacent genes are often co-regulated by the same transcription factor (TF) (5). In the same spirit, Janga *et* *al*. discovered that the majority of TFs exhibit a strong preference to regulate genes on specific chromosomes (6). Moreover genome-wide studies of expression data in several organisms revealed that genes from the same genomic neighborhood tend to have similar expression (7–9). Tendency of interacting proteins to aggregate on chromosomes was observed in yeast (10,11). The clustering trend was observed also in pathways, where Lee and Sonnhammer investigated the levels of clustering within pathways in five eukaryotic species, and found that a large fraction of the pathways exhibits significantly higher clustering levels than expected by chance (12). The aforementioned studies along with a handful of others indicate that there is a link between the relative genomic position of genes and their functional relations, though the eukaryotic clusters are usually much less compact than their prokaryotic counterparts (13). This relatively weaker clustering effect may imply that a more complex mechanism underlies gene arrangement in eukaryotes, incorporating a diversity of influences from multiple types of functional relations.

Furthermore, throughout the past decade it has become clear that the spatial arrangement of genes within the nucleus is also non-random (14). Folding and intermingling of chromosomes may result in high proximity between genes located at distant positions along the genome, including genes from different chromosomes. It was observed that while gene-rich chromosomes in human tend to occupy interior positions in the nucleus, their gene-poor counterparts tend to be peripherally located (15). Several studies have shown that transcription occurs within discrete regions known as transcription factories (16,17) and nuclear speckles (18). Moreover, evidence for co-expression of spatially proximal genes has been accumulating (19–21).

In this study, we develop a general methodology for analyzing the connection between functional gene groups and the linear and spatial arrangement of genes in the human genome. We focus on three types of functional groups: Protein–protein interactions (PPIs), complexes and pathways. We analyze three different facets of gene arrangement: the tendency of genes from the same group to concentrate on few chromosomes, the intra-chromosomal proximity of genes from the same group, and the degree to which genes from the same group tend to lie close to each other in the three dimensional (3D) space within the nucleus. Our findings show that functionally related genes tend to co-localize and manifest clustered organization within and across the chromosomes on all three levels.

## MATERIALS AND METHODS

Throughout the paper, we shall use the general term *group* to denote a single functional unit from any type, i.e. a PPI, a complex or a pathway. Note that each type reflects a relation of a different nature: The two members of a PPI are in direct physical contact under some conditions, while complex members are simultaneously involved as building blocks in the same physical unit. In contrast, pathways summarize sequences of multiple chemical or signaling reactions, and hence some of their members may not physically interact, co-localize or even simultaneously exist.

### Human Data

The Human PPIs, complexes and pathways analyzed in this study were taken from IntAct (22), Corum (23) and KEGG (24) respectively. Basic information about the three types of datasets that were used in the analysis is summarized in Table 1.

**Table 1.** Statistics on the group types used

Database	Functionality relation	Number of groups	Group sizes			Total number of genes involved
			Min	Median	Max	
IntAct	PPIs	27 947	2	2	2	7669
Corum	Complexes	1512	2	3	142	2421
KEGG	Pathways	206	2	49	1079	4852

Chromosomal locations of genes were extracted from NCBI MapView, where only protein coding genes with a unique position were kept. This preliminary filtering resulted in 19 287 genes.

Spatial distances between genes were based on the Hi-C experimental data of human lymphoblastoid cell line GM06990 (25). The 3D similarity matrices normalized by (26) were used.

### Removal of Tandem Duplicate Genes

Duplicate genes are expected to have similar functionality by ancestry. Such genes, if generated by tandem duplication, are often located in physical proximity to one other. To avoid clustering effects resulting from tandemly duplicated genes, we eliminated them as done in previous studies (8,9,27), in the following way. First, to identify proteins belonging to the same gene family, an all-against-all BlastP search was performed on all proteins in the genome, and families were defined using the MCL software (28) with default parameters. We then merged consecutive genes of the same gene family. The location of a merged gene was set to the interval spanning the consecutive genes that it replaced. The resulting set contained 18 029 genes.

### Statistical Methodology

In order to investigate whether genes from the same group tend to preferentially lie in proximity to each other, we created several tests, each examining a different form of co-localization. The *P*-value calculation for each of the tests is performed as follows:

- Formulate a test statistic that measures the proximity between functionally related genes.
- Calculate the value of this statistic, v_0_, for the real genome.
- Estimate the probability to observe this value or higher (alternatively, smaller) for random gene order.
  - Randomly permute the locations of genes to create a genome with random gene order (functional groups are unchanged).
  - Calculate the statistic value, v, for the resultant genome.
  - Repeat steps (iii)(a) and (iii)(b) *n* times.
  - Calculate the number of times *k* in which v ≥ v_0._
  - *P*-value = (*k* + 1)/(*n* + 1).

The random permutations used to create the null model ensure that the genes in the resultant genomes lie only in loci occupied by genes in the real genome, and that the number of genes in each chromosome remains unchanged. Moreover, the gene composition of the functional groups is unaltered. In this way we exclude from our null hypothesis effects that are not related to the gene order itself. We used the Bonferroni correction whenever multiple tests were performed.

Our tests collect information regarding the distribution of values we are interested in, e.g. how many groups are concentrated in *k* chromosomes, or what is the distribution of distances along the chromosomes—or in 3D space—between genes from the same group. The most natural test statistics are the moments of the distribution. However, sometimes we need a more sensitive test that focuses on the concentration at the tail of the distribution. In the *distribution tail test*, values are measured and partitioned into bins $b_1 ,b_2 , \ldots ,b_k$ where the frequency *f_i_* of values in bin *b_i_* is calculated. The test measures the extent of concentration in the first few bins. We seek the minimal number *j* for which the cumulative frequency $f_1 + \cdots + f_j$ is significantly higher than expected at random (see more details in the Supplementary Information). The *P*-value was calculated by comparing the real-genome cumulative histogram as in step 3 above, and Bonferroni corrected via multiplying by *j*.

## RESULTS

**Figure 1.**
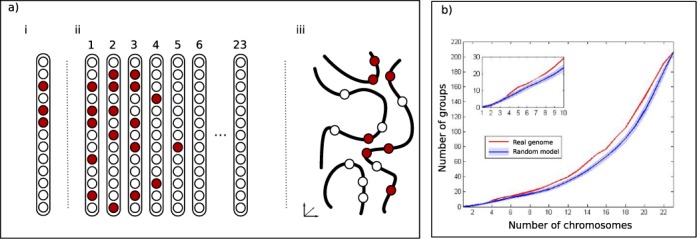
Proximity criteria and pathway concentration. (**a**) Criteria for chromosome concentration of co-functioning genes. Circles correspond to genes and the red circles are all the members of a group with a common function. (i): concentration within a chromosome (intra-chromosomal). Here clustering/concentration is gauged based on pairwise linear distance (in base pairs or in the number of intervening genes) between co-functioning genes. (ii): dispersal across chromosomes (inter-chromosomal). Out of the 23 pairs of chromosomes, 18 do not contain the group's genes, so this group is concentrated in few chromosomes. This measure takes into account only the chromosomes on which the co-functioning genes reside and ignores the relative locations within each chromosome. (iii): concentration in the 3D space. Curved lines show positions of chromosomal segments in space, with the genes on them. The group is concentrated in space. By identifying the chromosome each segment belongs to, one can distinguish between proximity of inter- and intra-chromosomal gene pairs, and analyze them separately. (**b**) Pathway concentration in few chromosomes. For each number *j* of chromosomes, the plots show the number of pathways whose genes reside in at most *j* chromosomes. Plots for the real genome (red curve) and for an average over 10^6^ random genomes (blue curve) are shown. The shaded area around the blue curve shows ±1 standard deviation. Inset: Zoom in on the region of a small number of chromosomes.

We set out to test the tendency of genes with a common function to cluster in the genome using three complementary measures (Figure 1a): inter-chromosomally, by measuring the number of chromosomes co-functioning genes are distributed on; intra-chromosomally, using the genomic distances between co-functioning genes, and in 3D space, by measuring the proximity in the nucleus between co-functioning genes. These three approaches are complementary and each addresses a different aspect of the concentration.

### Inter-chromosomal dispersal of genes with a common function

We first investigated the tendency of genes from the same group to concentrate on a small number of chromosomes. Abstractly, each functional type (PPI, complex of pathway) is a collection of groups of genes, where each group shares a common function. For each group, we defined the number of chromosomes involved in the group as the number of different chromosomes containing genes from that group. Our first test function was defined to be the average of this number over all groups of the same type. We generated 10^6^ random genomes by randomly permuting the locations of the genes, and calculated a *P*-value for each of the group types using the procedure described in ‘Statistical Methodology’ section. The results show that for both PPIs and pathways, the groups tend to concentrate on a small number of chromosomes with *P*-values 0.001 and $ \le 10^{ - 6}$ respectively. This test yielded no significant results for complexes (*P*-value 0.08).

In light of these positive findings, we proceeded with a higher resolution examination of gene arrangement into chromosomes, and applied the distribution tail test. Here *f_i_* is defined as the number of groups involving *i* chromosomes, in order to measure the extent to which genes from the same group tend to concentrate on few chromosomes. The *P*-value was calculated by comparing the real-genome frequencies to those in 10^6^ random genomes. The results show that there is an enrichment of PPIs and complexes involving a single chromosome (*P*-value = 0.001 and 0.01, respectively), i.e. the number of PPIs and complexes all of whose genes reside on a single chromosome is significantly higher than randomly expected.

In the case of pathways we reveal a similar trend. The number of pathways that are represented on at most *c* chromosomes is exhibited in Figure 1b. For *c* ≥ 5, this number is significantly higher than expected at random (*P*-value = 0.03 after Bonferroni correction). Hence we observe a tendency of pathways to concentrate on fewer chromosomes than expected by chance.

### Intra-chromosomal distances of co-functioning genes

In the previous section, we checked whether genes from the same functional group tend to concentrate on fewer chromosomes than expected by chance. In this section, we would like to check whether genes from the same group that belong to the same chromosome tend to be closer than expected. In order to measure this clustering tendency, we calculated for each group *i* the average distance (in bases), *d_i_*, between pairs of genes in group *i* that reside on the same chromosome. We defined our test statistic to be the mean value of *d_i_* over all groups in the dataset (see Supplementary Information for more details). We used 10^6^ random genomes to calculate the *P*-values based on our statistical methodology, where this time we randomly permuted the locations of genes within each chromosome separately. In this way we accounted for clustering effects due to concentration of genes from the same group on few chromosomes. The results show that the average intra-chromosomal distance between genes from the same complex and pathway is significantly smaller than expected by chance, obtaining *P*-values of 0.001 and $ \le 10^{ - 6}$ respectively. No significant result was obtained for PPIs (*P*-value = 0.15).

**Figure 2.**
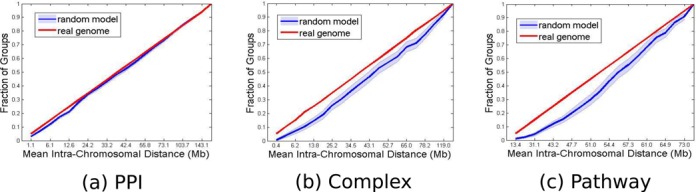
Intra-chromosomal distances. The plot shows the average intra-chromosomal distance between genes from the same group in the real (red) and randomized (blue) genomes. (**a**) PPIs; (**b**) Complexes; (**c**) Pathways. Bins were selected so that the occupancy of pairs from the real genome is uniform (hence the straight red line). The clustering effect is reflected by the larger cumulative fraction in the real genome histograms compared to the random model in the smaller distance bins. The light blue shaded region around the blue curve stands for ±1 standard deviation.

Next, we conducted the more sensitive distribution tail test, focusing on groups with short average distances between members. We partitioned the gene groups into 20 bins based on the distances *d_i_* defined above, as follows. The distances were sorted, and thresholds $0 = t_0 < t_1 < \cdots < t_{20}$ were set such that 5% of the distances were between $t_{i - 1} \;{\rm and}\;t_i$ (see Supplementary Information for more details). We then used these thresholds to bin distances for each of 10^5^ random genomes defined as above. We used a lexicographic order of bin frequencies to refine the results (see Supplementary Information). We discovered that for all group types, a statistically significant number of groups tend to cluster within smaller distances than expected at random (all three with *P*-values $ \le 10^{ - 5}$, Bonferroni corrected). This tendency is illustrated in Figure 2. The cumulative distributions of the true genome are plotted along with their random counterparts obtained by averaging over the 10^5^ random histograms. The figure shows the enrichment of short distances for each of the three functional group types.

### Spatial arrangement of co-functioning genes

In this section we analyze the spatial proximity of functionally related genes in the nucleus. The spatial distances that we used were based on contact map data generated by Lieberman-Aiden *et* *al*. using the Hi-C technology (25) and renormalized by Yaffe and Tanay (26) (see Supplementary Information for details). The contact map gives the frequency of observing each two genomic segments next to each other in the experiment. Segment sizes were 1 Mb. As in (25), correlation between the frequency vectors of segments was used to measure proximity, and we use 1-correlation as a measure akin to 3D distance.

We first applied the distribution tail test as follows. For each group we computed the average distance between pairs of genes in the group, irrespectively of whether they reside on the same chromosome or on different chromosomes. The results show that for all three types of groups, functional gene groups exhibit more spatial concentration than expected at random (*P*-value = 10^−4^, calculated from 10^4^ simulations). However, the high correlation between linear intra-chromosomal distances (as measured in base pairs) and the corresponding 3D distances (see Supplementary Figure S1 in Supplementary Information) raises the question whether the apparent 3D concentration is merely a result of the linear intra-chromosomal concentration observed earlier. In order to test for spatial concentration effects that are not related to linear gene proximity, we *considered only inter-chromosomal gene pairs*, excluding all intra-chromosomal distances. To respect the chromosomal organization of each group, we again randomly permuted the locations of genes within each chromosome separately. So, for each group the number of pairs of genes along different chromosomes stays the same in simulated genomes. The results show that the average inter-chromosomal 3D distances between genes from the same pathway are significantly smaller than expected by chance (*P*-value = 0.009; complexes *P*-value = 0.09, PPIs *P*-value = 0.7). Applying the distribution tail test with 20 bins resulted in significant over-population of the first bin for PPIs only (*P*-value = 0.004). For complexes and pathways, *P*-values were 0.06 and 0.33 respectively.

**Figure 3.**
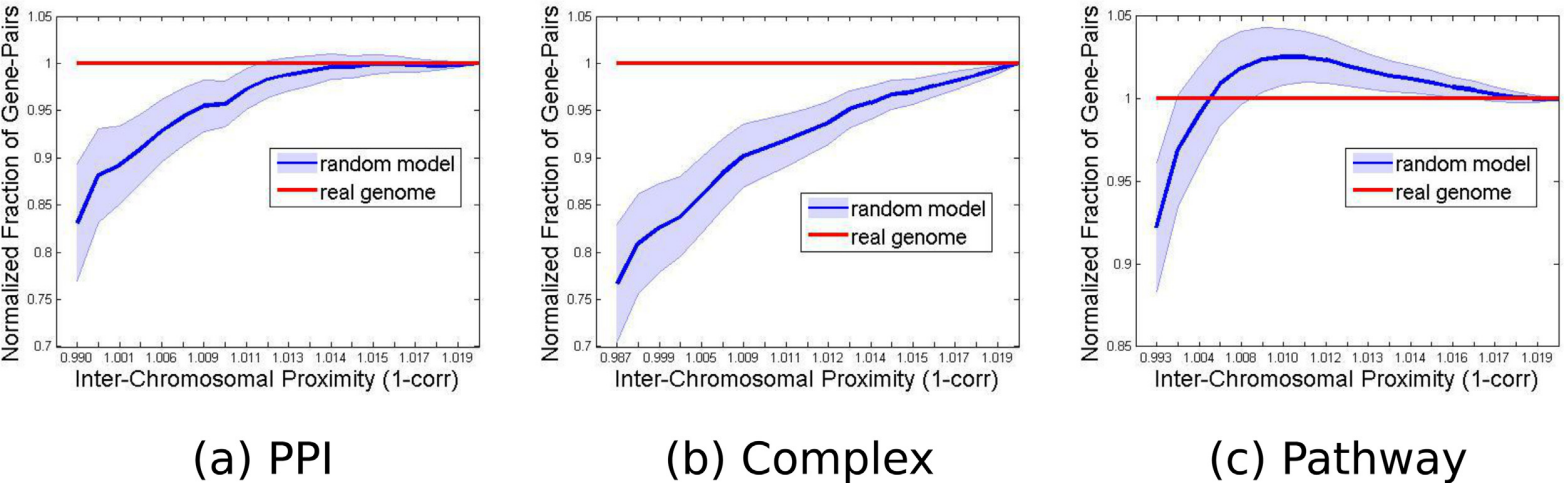
Spatial proximity between inter-chromosomal gene pairs from the same functional group. The cumulative distribution function (cdf) of inter-chromosomal proximity between genes from the same group was computed for real and randomized genomes. The plot shows each cdf divided by the cdf of the real genome. As a result the real genome curve has a constant y-value of 1. Red: real genome, blue: average over 10^5^ random genomes. The light blue bands show ±1 standard deviation. (**a**) PPIs; (**b**) Complexes; (**c**) Pathways. The x-axis units are 1 minus the correlation between the normalized Hi-C contact profiles of the regions containing the gene pairs, so that smaller values reflect higher correlation and shorter distances.

The distribution tail test used above checks whether the *average* inter-chromosomal distances between gene pairs within a group is significantly smaller than expected at random. However, if proximity tendency exists only between specific genes within a group, it may be undetected after averaging all pairs in the group. This could explain the fact that the test was significant for PPIs but not for the other types, which have larger groups. To examine whether such tendency exists, we applied the distribution tail test again, but this time we used the individual distances between gene pairs instead of the average over these distances. We computed the distance between each pair of genes from the same group that reside on different chromosomes, binned the values obtained from the entire set of such pairs into 20 bins, and tested the concentration at the distribution tail. We found that for all three group types, namely PPIs, pathways and complexes, gene pairs from the same group tend to cluster within a small spatial region even when they lie on different chromosomes. For the three resultant distributions, the first bin in the real genome (5% of pairs with highest spatial inter-chromosomal proximity) was significantly more populated than the same bin in the random genomes, with *P*-values 0.004, 10^−4^ and 0.02 for PPIs, complexes and pathways respectively. This result reflects the clustering tendency of genes from the same group. For PPIs and complexes, the cumulative distribution of the histogram tail remained statistically significant also beyond the first bin. For PPIs, more than 25% of the pairs displayed strong clustering tendency (e.g. for the sum of frequencies in bins 1–6, the obtained *P*-value was ≤0.03). An even more pronounced effect was found for complexes, where about 90% of the pairs, populating bins 1–18 in the cumulative histogram, had all *P*-values below 0.02. These results, normalized by dividing by the real genome values for the sake of better visibility, are illustrated in Figure 3.

All the tests that we conducted aimed to deduce concentration by looking together at the signal from all groups of co-functioning genes of the same types. As such, they provide answers regarding the general phenomenon of concentration. In addition, our analysis can also be applied to study the concentration of individual groups, which may be of independent interest. Supplementary file 1 contains the full results of each PPI, complex and pathway in each of our three tests. We also tested functional categories of complexes for concentration but obtained no significant results (see Supplementary Information).

## DISCUSSION

We have observed that co-functioning genes manifest significant concentration in terms of their organization in the human genome. This holds separately for three types of sets of co-functioning genes (Table 1): gene pairs corresponding to interacting proteins, genes whose proteins belong to the same complex, and genes whose proteins take part in the same pathway. The concentration of co-functioning genes is established in three independent ways (Figure 1a). Co-functioning genes tend to reside on fewer chromosomes than expected by chance. When they are on the same chromosome, they are positioned more closely to each other than randomly selected genes. Moreover, co-functioning genes on different chromosomes tend to be closer to each other in the 3D nuclear space, based on chromosome conformation capture (3C (29) or Hi-C) data.

These tendencies are statistically significant, based on a cumulative signal collected from many groups of co-functioning genes. The distribution of the test statistic (e.g. the spatial distance or the number of involved chromosomes) in the known functional groups is compared to randomly permuted genes (within and/or across chromosomes, where appropriate). In some cases a simple test statistic, like the distribution mean, suffices to determine significance. In others, we tailored a test statistic to focus on the tail of the distribution, corresponding to the closest pairs.

Why are co-functioning genes concentrated? The broadly accepted explanation has to do with co-transcription. Co-location of genes of common function can facilitate direct cis-regulation of several genes simultaneously, at the level of transcription factors and co-factors, and on the nucleosome and other epigenetic levels. Caron *et* *al*. observed clustering of highly expressed genes on intervals along the chromosomes in a variety of human tissues (30). Lercher *et* *al*. later observed the same phenomenon for housekeeping genes, and in fact argued that the findings of Caron *et* *al*. are due primarily to housekeeping genes (9). In lower eukaryotes, by focusing on two or at most three consecutive genes, the co-regulation of adjacent divergent transcriptional units was shown to be prevalent in yeast (5). Taking an evolutionary perspective, Veron *et* *al*. analyzed chromosomal rearrangements between mouse and human and showed significant correlation between intra-chromosomal 3D proximity in the human genome and breakpoint pairs, suggesting the functional relevance of the structure (31). Another evolutionary analysis was recently provided by Dai *et* *al*., using gene orders in 17 yeast species (32). The authors showed that gene pairs that are adjacent in other yeast species but reside on different chromosomes in *Saccharomyces cerevisiae* tend to show stronger nuclear co-localization, as measured in (33). Moreover, these co-localized pairs tend to be regulated by the same transcription factors and by the same histone modifications. Hence co-localization is correlated with co-regulation even after separation due to recombination.

The connection between spatial organization within chromosomes and gene expression was attributed to active chromatin hubs (34), nuclear speckles (18) and more generally to transcription factories. These are discrete nuclear regions in which multiple RNA polymerases are active (35). However, evidence for and against the existence of transcription factories is still debated (36). Li *et* *al*. recently studied extensively chromatin interactions in human cell lines and observed promoter–promoter interactions, to the extent that they proposed a chromatin-based operon-like mechanism (‘chroperon’) for gene regulation in eukaryotic cells (37). Co-expression and proximity in space were shown to be associated both in studies focusing on a few genes using FISH and microscopy, and, more recently, in genome-wide studies of promoter-enhancer associations (38,39). One novel perspective that we add to this area is the inter-chromosomal dispersion: we show that genes of a common complex or pathway tend to be dispersed on fewer chromosomes than expected by chance. With such clustering, co-regulation of the co-functioning genes is conceivably better than when the dispersal is completely random.

Is it possible that we see co-clustering of members of the same functional gene groups only because they have similar expression levels? Put differently, is the primary phenomenon co-expression of genes of the same functional group, and the co-localization is only its secondary effect? While it is hard to say which effect is primary, co-expression and common function clearly both affect co-localization. There are, however, several advantages to analysis based on common function over analysis based on co-expression: (i) Sharing the same functional group is a ‘cleaner’ and more universal property than co-expression, which is measured on condition-dependent datasets. (ii) Co-expression quantification is based on measurements of expression, which are noisy. (In fact, co-expression may even be the result of biological noise, cf. (40)). Moreover, gene transcription, the main source of co-expression measurements today, is only moderately correlated with protein transcription, where the function is manifest (41,42). (iii) Different pathways or complexes may show co-expression under some conditions even if they have completely different functions. (iv) Since many pathways summarize a temporal sequence of events and interactions, different segments of the pathway may be active at different times and in that case will not show co-expression, even though they belong to the same functional unit. (v) The definition of a group of co-expressed genes can vary depending on the correlation function, the correlation threshold, the normalization methods etc. On the other hand, functional groups are defined based on a holistic understanding of the underlying biology. (vi) Our analysis enables us to examine different types of co-functioning groups, and discern differences among them, which is impossible using co-expression. Further study is required to show which effect is more primary, or that perhaps both co-expression and co-localization are artifacts of yet another more basic, global effect.

The study of nuclear organization has undergone a revolution over the last decade, with the combined contribution of microscopy techniques, chromosome conformation and epigenomics. Seminal studies have established chromatin proximity maps in human (25), baker's yeast (33), fission yeast (43), drosophila (44) and mouse (45), among others. Very recently, an interesting paper by Ben-Elazar *et* *al*. (46) studied localization of co-regulated genes in *S. cerevisiae* using 4C data (33). Focusing on the set of targets of each transcription factor, the authors showed that for about half the transcription factors, the concentration of these targets in space exceeds their linear clustering along the chromosomes. This adds support to the transcription factories paradigm. Note, however, that the statistical test for 3D concentration does not distinguish between targets that are on the same chromosome and those on different chromosomes. In fact, the intra-chromosome contact level observed in 3C maps exceeds the inter-chromosomal level by orders of magnitude (25,33), and therefore it is highly likely that the effect observed by Ben-Elazar *et* *al*. is based overwhelmingly on intra-chromosomal contacts. In contrast, our test (Figure 3) separates completely the inter- and intra-chromosomal signals, and shows directly inter-chromosomal spatial proximity among co-functioning genes. Another recent paper (47) analyzed the same yeast contact map data (33) together with gene expression data. Using a large panel of expression profiles, and focusing on inter-chromosomal gene pairs only, Homouz and Kudlicki showed that the measured expression levels of nearby genes are significantly correlated. Moreover, they showed that many of the high level gene ontology groups (GO-slim groups) show significantly more 3D contacts between gene pairs than random gene groups of the same sizes. This test is similar in spirit to the one we have performed here. Note, however, that we took care to create gene sets by randomly permuting genes within each chromosome independently, thereby avoiding possible bias due to uneven distribution of group genes across chromosomes. Another salient difference between our study and these two reports is that our test was conducted on human DNA, for which the contact map resolution is much lower than for yeast, and hence detecting the concentration signal is more challenging. To the best of our knowledge, this is the first study of this kind on the human chromosomal conformation data.

In Figure 3, the normalized cumulative distributions for randomized genomes remain below the real genome plot for PPIs and complexes, showing spatial concentration of co-functioning genes. For short distances this is the case for pathways as well, but for longer distances the real genome apparently has fewer pairs than the randomized genomes. A possible tempting explanation may be due to the different nature of the functional groups. PPIs and complexes consist of proteins that are simultaneously interacting, and thus their co-location (and consequently co-expression) may be an advantage. In contrast, for pathways a time dimension is involved (e.g. when a sequence of metabolic or signaling reactions is performed), and therefore not all the building blocks of the pathway may be active simultaneously. In a large pathway it may be favorable for subunits that act together to be closer in space, and subgroups that act at different times to be well separated in space. Since many of the pathways that we have analyzed are rather large (median size 49), they may contain such non-simultaneous blocks that may give rise to their distinct distribution. This hypothesis requires further analysis and testing.

Gene clusters and tandem gene duplications can affect our intra-chromosomal statistics. In order to remove such effects, we removed known gene clusters and also filtered tandem duplicated genes as done previously (8,9,27). It is possible though that part of the effect that we observe is a result of unknown clusters or remaining duplicated genes that do not appear in tandem. However, this would not explain the inter-chromosomal spatial concentration.

Finally, the test methodology that we developed here can be useful for studying other questions as well. It provides a unified approach for comparison of true and random distributions for a broad variety of test statistics.

## SUPPLEMENTARY DATA

Supplementary Data are available at NAR Online.

SUPPLEMENTARY DATA
