# Supplementary material for: Functional gene groups are concentrated within chromosomes, among chromosomes and in the nuclear space of the human genome
Source: Nucleic Acids Res. 2014 Jul 23;42(15):9854–61. doi: 10.1093/nar/gku667 (PMC4150778; doi:10.1093/nar/gku667)
Supplement: SUPPLEMENTARY DATA [file supp_42_15_9854__index.html]

Functional gene groups are concentrated within chromosomes, among chromosomes and in the nuclear space of the human genome — Functional gene groups are concentrated within chromosomes, among chromosomes and in the nuclear space of the human genome — SUPPLEMENTARY DATA 

# HIV-1 Nef and KSHV oncogene K1 synergistically promote angiogenesis by inducing cellular miR-718 to regulate the PTEN/AKT/mTOR signaling pathway

## SUPPLEMENTARY DATA

**Files in this Data Supplement:**

- SUPPLEMENTARY DATA 2
- SUPPLEMENTARY DATA 1
