# Supplementary material for: Functional gene groups are concentrated within chromosomes, among chromosomes and in the nuclear space of the human genome
Source: Nucleic Acids Res. 2014 Jul 23;42(15):9854–61. doi: 10.1093/nar/gku667 (PMC4150778; doi:10.1093/nar/gku667)
Supplement: SUPPLEMENTARY DATA [file supp_gku667_nar-01446-z-2014-File005.docx]

# Thévenin et al. Functional gene groups are concentrated within chromosomes, among chromosomes and in the nuclear space of the human genome

Supplementary Information

Data

**Human genome**

For gene location in the Human genome, we used the July 2011 versions of NCBI^[[1]](#footnote-1)^ files Homo_sapiens.gene_info.txt^[[2]](#footnote-2)^ for the curated genes, and seq_gene.md^[[3]](#footnote-3)^ for the genes’ base position. Among the 42,158 entries of Homo_sapiens.gene_info.txt, we selected the set of 20,329 genes annotated as protein coding and present in a homologous chromosome. Among the 1,534,264 entries of seq_gene.md, we selected the set of 36,429 annotated as protein coding genes, and according to the GRCh37.p2-Primary assembly, present in a homologous chromosome with only one known base position. Then, we defined the set of 19,287 genes whose chromosome was the same in and . In this way, all genes in had a single gene ID in Entrez. Genes from chromosome Y were excluded from the analysis.


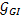

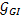

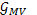

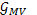

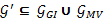

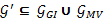

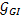

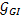

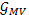

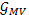

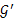

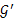


Finally, from we obtained a set of size 18,029 by merging tandem duplicated genes into a single gene as described in the Methods section.


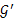

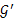

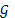

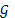


**Co-functioning genes**

For PPIs we used the IntAct database (July 2011 version). For pathways, we used KEGG (30 June 2011 version). For complexes, we used CORUM (September 2009 version). In order for a functional gene group to be included in our analysis the following conditions were set:

- The group is unique,
- It has at least two different genes in ,


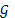

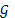


- At least 95% of the genes in the group are in ,


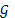

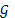


- The group has at most one gene from each known gene cluster (Hox genes, olfactory receptor genes, Human Leukocyte Antigen (HLA) genes and Hemoglobin genes).

The last constraint removes the influence of large known gene clusters from our analysis.

In IntAct, the genes in each group were given by their UniProt^[[4]](#footnote-4)^ identifier which we converted to gene IDs using the file HUMAN_9606_idmapping.dat^[[5]](#footnote-5)^. PPIs with at least one protein that had either no geneID match or several matches were ignored.

**3D Human genome**

Lieberman-Aiden et al. used the Hi-C method to study the three-dimensional architecture of a whole genome by coupling proximity-based ligation with massively parallel sequencing (1). They constructed spatial proximity matrix of the human lymphoblastoid cell line GM06990 genome, based on contact probability between genomic regions at a resolution of 1Mb. In order to reduce biases, we adopted the normalization method proposed by Yaffe and Tanay (2) , which was shown to outperform the original one of Lieberman-Aiden et al.. A later method of Imakaev et al. (3) was reported to yield essentially the same matrix of biases. Because the extremities of chromosomes are not included in the proximity matrix, some genes do not have spatial measurements. As a result, groups containing less than 2 genes with spatial measurements are ignored during our the 3D proximity tests. This excluded 426 PPIs (among 27,947) and 5 complexes (among 1,512).

Methods

**Inter-chromosomal dispersal of genes with a common function** - **the distribution tail test**

Let be the number of groups involving exactly chromosomes in genome , and let be a vector representing the total number of groups involving at most chromosomes, namely, the *i*-th component of is given by Denote the number of chromosomes by . To calculate the relative fraction of random distributions that are at least as concentrated as the real one, we use the following definition: Given two frequency vectors and , we define to be more concentrated than starting from bin , iff is lexicographically larger than starting from bin , i.e., there exists , , such that and . Clearly, the two histograms are equally concentrated if and only if .


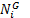

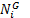

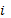

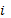

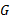

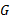

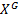

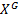

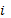

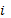

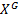

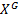

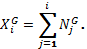

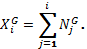

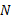

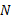

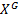

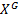

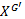

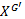

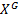

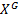

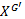

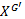

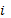

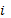

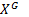

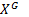

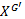

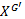

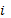

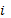

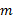

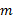

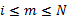

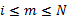

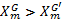

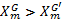

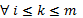

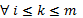

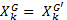

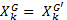

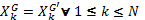

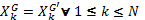


**Testing intra-chromosomal distances between co-functioning genes**

Define the distance between two genes on the same chromosome as the number of base pairs between the last base of the first gene and the first base of the second gene. Let be the average distance between all pairs of genes from the same chromosome involved in group . (Note that the average can include different pairs from different chromosomes).


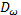

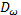

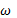

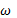


- **The average test:** The average test function is defined to be the average of over all groups of the same type.


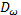

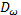

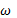

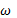


- **The distribution tail test:** The distances for the real genome were sorted, and thresholds were set such that 5% of the distances were between and , i.e., in bin . More precisely, when the number of distances did not allow the bins to be evenly populated, the first bins were populated with groups each, and the last bin with the remaining groups. Moreover, cases where multiple groups had the same average distance as the bin threshold often resulted in slight differences between the bin populations. For genome let be the number of distances that fall in bin . By generating many randomized genomes and recording their vectors, the significance of the concentration of the values within the first few bins in the real genome can be evaluated. Starting from the first bin we perform a sequence of tests with the test functions to find the first for which a significant p-value (after Bonferroni correction) is obtained.


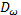

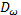

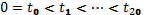

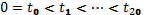

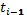

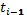

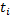

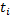

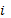

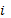

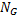

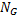

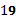

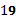

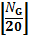

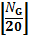

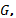

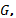

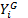

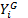

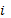

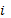

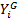

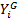

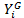

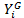

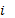

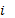


**Intra-chromosomal distances along the genome and in space**

We wanted to test the correlation between spatial and linear intra-chromosomal distances. For the spatial normalized matrix, we computed the Pearson correlation of each pair of rows. This computation yielded, for each pair of 1Mb chromosomal regions and , a value between -1 and 1. For every pair of genes, and , residing on regions and respectively, we defined the distance between them to be (if a gene is spread over several different regions, we take the weighted average of according the number of bases present in each region). The 265 genes located on regions that were not included in the spatial proximity matrix from (1) were removed from our analysis. **Figure S1** shows the high correlation between the spatial and the linear measures, at short intra-chromosomal distances**.**

Figure S1 Relation between intra-chromosomal linear distance (number of bases) between pairs of genes and the spatial proximity between them (represented by 1-correlation), as measured in (1, 2).

**Testing the association between functional categories of complexes and inter-chromosomal 3D distances**

**We conducted the following additional test to see if certain functional categories of complexes are significantly concentrated in 3D.**

1. Filter out any gene that is not covered by the Hi-C data.
   Filter out complexes whose number of genes is zero or one after genes filtering.
   [5 complexes were filtered out due to missing 3D data.]
2. Order all inter-chromosomal gene-pairs that are found within complexes by their proximity in 3D (overall there were 31648 such pairs)
3. For a tested functional category of complexes, derive the set of inter-chromosomal gene pairs appearing in its complexes. Call this set S.
4. Test whether S is enriched with low/high 3D distances by a GSEA test (4, 5) and obtain an enrichment score ES(S). To compute its significance, apply the following procedure:
   - 1. Randomly permute all genes within their chromosomes
     2. Rank inter-chromosomal gene pairs by the 3D-proximity values corresponding to their new locations
     3. Recompute the enrichment score of S on the resulting set.

Report the empirical p-value of ES(S) obtained by 1000 runs of the procedure

We used MIPS FunCat for functional annotation scheme of CORUM complexes, covering 1512 complexes. We applied the test described above to the 23 classes in the highest level in this annotation hierarchy. Table S1 below presents the results of the test. None of the tested functional categories was found to be significantly enriched with lower 3D values after Bonferroni correction for multiple testing.

Table S1: Significance results for enrichment of MIPS functional categories in inter-chromosomal 3D distances

1. Lieberman-Aiden, E., Berkum, N.L. van, Williams, L., Imakaev, M., Ragoczy, T., Telling, A., Amit, I., Lajoie, B.R., Sabo, P.J., Dorschner, M.O., et al. (2009) Comprehensive Mapping of Long-Range Interactions Reveals Folding Principles of the Human Genome. *Science*, **326**, 289–293.

2. Yaffe, E. and Tanay, A. (2011) Probabilistic modeling of Hi-C contact maps eliminates systematic biases to characterize global chromosomal architecture. *Nat. Genet.*, **43**, 1059–1065.

3. Imakaev, M., Fudenberg, G., McCord, R.P., Naumova, N., Goloborodko, A., Lajoie, B.R., Dekker, J. and Mirny, L.A. (2012) Iterative correction of Hi-C data reveals hallmarks of chromosome organization. *Nat. Methods*, **9**, 999–1003.

4. Mootha, V.K., Lindgren, C.M., Eriksson, K.-F., Subramanian, A., Sihag, S., Lehar, J., Puigserver, P., Carlsson, E., Ridderstråle, M., Laurila, E., et al. (2003) PGC-1α-responsive genes involved in oxidative phosphorylation are coordinately downregulated in human diabetes. *Nature Genetics*, **34**, 267–273.

5. Subramanian, A., Tamayo, P., Mootha, V.K., Mukherjee, S., Ebert, B.L., Gillette, M.A., Paulovich, A., Pomeroy, S.L., Golub, T.R., Lander, E.S., et al. (2005) Gene set enrichment analysis: A knowledge-based approach for interpreting genome-wide expression profiles. *PNAS*, **102**, 15545–15550.

1. http://www.ncbi.nlm.nih.gov/ [↑](#footnote-ref-1)
2. ftp://ftp.ncbi.nih.gov/gene/DATA/GENE_INFO/Mammalia/ [↑](#footnote-ref-2)
3. ftp://ftp.ncbi.nih.gov/genomes/MapView/Homo_sapiens/sequence/BUILD.37.2/initial_release/ [↑](#footnote-ref-3)
4. http://www.uniprot.org/ [↑](#footnote-ref-4)
5. ftp://ftp.uniprot.org/pub/databases/uniprot/previous_release/releases-2011/knowledgebase/idmapping/by_organism/ [↑](#footnote-ref-5)
